# Hand function in gout: a comparative study with rheumatoid arthritis and healthy

**DOI:** 10.1590/1806-9282.20250054

**Published:** 2025-07-07

**Authors:** Oguzhan Mete, Hakan Apaydin, Fatmagül Varol, Emine Gözde Aydemir Gülöksüz, Melih Pamukcu

**Affiliations:** 1 University of Health Sciences, Gülhane Faculty of Physiotherapy and Rehabilitation – Ankara, Turkey.; 2 fztoguzhanmete06@gmail.com

**Keywords:** Gout, Arthritis, Hand, Grip, Functional status

## Abstract

**OBJECTIVE:**

The aim of the study was to compare the hand function (grip strength and grip endurance, manual dexterity, and perceived hand functional disability) of participants with gout, rheumatoid arthritis, and healthy control.

**METHODS:**

Grip strength and grip endurance were assessed using a hand dynamometer, while manual dexterity was evaluated through the Nine-Hole Peg Test. Perceived hand functional disability was measured using the Duruöz Hand Index.

**RESULTS:**

The grip strength was similar between gout and healthy control (p>0.05). Dominant and non-dominant grip endurance in gout were lower than healthy control (p=0.008, p=0.001). The dominant placing, removing, and total Nine-Hole Peg Test time of gout compared to healthy control were higher (p=0.001, p<0.001, and p<0.001). The non-dominant placing, removing, and total Nine-Hole Peg Test time of gout were higher than healthy control (p=0.002, p=0.004, and p=0.002). The Duruöz Hand Index in gout was higher than healthy control (p=0.002), but lower than rheumatoid arthritis (p=0.008).

**CONCLUSION:**

Our findings indicate that individuals with gout experience impairments in grip strength, manual dexterity, and perceived hand function. Assessing hand function during clinical examinations of patients with gout should be considered.

## INTRODUCTION

Gout is characterized by the deposition of monosodium urate crystals in the synovial fluid and environmental tissues due to disturbed uric acid metabolism. Chronic gout arthritis leads to degenerative joint damage, soft tissue inflammation, tophus formation, and altered structural integrity of tendons and ligaments that may cause physical functional disability and a decrease in quality of life^1,2^. Studies examining the gout patients’ perspectives reported that gout can cause limitations in daily activities due to physical functional impairments^3^.

Hand function is crucial for performing daily activities independently and carrying out tasks requiring fine skills^4^. Grip strength (GS), which defines the strength of the hand muscles used to grasp or grip, is an important part of the functional activity of the hands. Besides, many basic activities consist of an endurance component, and when grip endurance (GE) is impaired, it can lead to varying degrees of disability and activity limitations^5^. Manual dexterity, manipulating objects with skillful, controlled arm-hand movements under speed conditions, requires a tremendous sensorimotor function. Coordinating the hand function with appropriate speed and precision, is important^6^.

Clinical manifestations may cause long-term functional disorders in gout^7,8^. Although pathologic signs of hand involvement in gout are reported, clinical research examining the hand function of gout patients is scarce^9,10^. Moreover, no study has compared the hand function of gout patients to rheumatoid arthritis (RA), a type of inflammatory arthritis that has been scientifically proven to have a significant impact on hand function^5^, and healthy control (HC). Therefore, we aimed to compare the hand function (GS and GE, manual dexterity, and perceived functional disability) between gout, RA, and HC.

## METHODS

### Design and sampling

This single-center study was designed as case-controlled research conducted by Ankara Etlik City Hospital Rheumatology Clinic between March and August 2024. The study was conducted in compliance with the ethical protocol approved by the Ankara Etlik City Hospital Clinical Research Ethics Committee (Date and Protocol Number: 31/01/2024-AEŞH-EK1-2024-0004) and strictly followed the Declaration of Helsinki.

Participants were diagnosed with gout according to the 2015 ACR-EULAR Gout classification criteria^11^, and diagnosed with RA according to the 2010 ACR/EULAR RA classification criteria^12^, and HC was involved. Participants were excluded from the study if they: (a) were unable to complete the assessment due to gout or severe RA flare; (b) had additional conditions affecting hand function; (c) had a history of upper extremity surgery or fractures; (d) had severe hand or wrist deformities affecting grip or pinch; (e) received a new diagnosis within the last 3 months; (f) were diagnosed with a psychiatric illness; or (g) had cooperation issues that hindered evaluation.

### Assessments

#### Demographic and physical characteristics and disease-related features

The age, body mass index (BMI), and sex were recorded for participants. The disease duration, medication, presence of gout flare, urate-lowering therapy, visible hand tophi, level of serum urate, creatinine, C-reactive protein (CRP), and erythrocyte sedimentation rate (ESR) of gout patients, and disease duration, medication, count of swollen and tender joints, level of CRP and ESR, and disease activity detected by disease activity-28 (DAS-28) for RA patients were recorded by their rheumatologist^13^.

#### Hand function

A hand dynamometer (Jamar Hand-Dynamometer, USA) was employed to evaluate the GS and GE. Participants were instructed to assume the standard test position as the American Hand Therapists Association recommended. They were then directed to squeeze the dynamometer with maximum force using their hands to assess the GS. Two trials were taken for each dominant and non-dominant side, with a 1-min interval between each measurement. The maximum value of two trials was then recorded^14^.

The GE test was performed in the same position as the GS test. During the GE test, the participant was asked to squeeze 50% of their maximum GS and maintain this value for as long as possible. The duration of the grip performance without any deviation from the targeted value was then recorded in seconds. The test was carried out for the dominant and non-dominant sides^14^.

The manual dexterity was assessed using the Nine-Hole Peg Test (NHPT). It contains a container including nine small pegs and holes. The test involves picking up nine small pegs from a container, placing them into a nine-hole grid, and returning them to the container. During the test, participants were asked to sit in front of a board and remove nine pegs from a container, place them into the holes, quickly remove them from the holes without stopping, and then put them back into the container. The test is performed using one hand as quickly as possible, and a shorter test time indicates greater manual dexterity. Test time can be calculated as the time taken for placing the pegs, the time taken for removing the pegs, and the total time^6^.

Functional disability of the hand was assessed using the Duruöz Hand Index (DHI). It consists of 18 questions in five main categories, including kitchen activities, dressing, cleaning, work-related tasks, and other daily activities. Each question is rated on a scale from 0 (no difficulty) to 5 (impossible). The scores from all five categories are combined to obtain a total score ranging from 0 to 90. Higher scores indicate greater functional disability or poorer hand function^15^.

### Statistical analysis

The required sample size for the study was calculated using G*Power Version 3.0.10, indicating that 72 participants (24 per group) were needed to achieve 90% power, with an effect size of f=0.436 and type I and II error rates of α=0.05 and β=0.10, respectively. Data analysis was conducted using IBM SPSS (Statistical Package for the Social Sciences) Statistics (Version 22.0). The chi-square test compared categorical variables, while one-way analysis of variance was used for normally distributed continuous variables. The Kruskal-Wallis test was applied when normality assumptions were not met. Significant results were followed by the Bonferroni correction, with statistical significance set at a p<0.05.

## RESULTS

**Figure 1:**
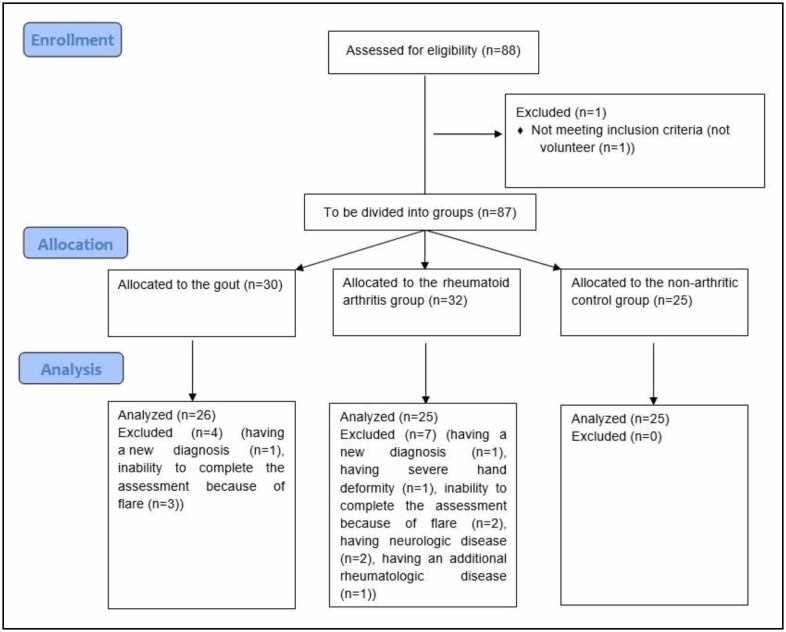
The flowchart of the study.

The flowchart of the study is given in Figure 1. The disease-related features are presented in Table 1. The age (p=0.019), BMI (p=0.059), and sex (p=0.912) were similar between groups.

**Table 1:** Disease-related features of participants with gout and rheumatoid arthritis.

		Gout group (n: 26)
Duration of diagnosis [months, median (IQR)]		70.0 (96.0)
Medication [n/26 (%)]		
	None	1 (3.8)
	Allopurinol	18 (69.2)
	Colchicine	21 (80.8)
Urate lowering treatment [n (%)]		
	Yes	18.0 (69.2)
	No	8.0 (69.2)
Presence of hand tophi [n (%)]		
	Yes	0.0 (0.0)
	No	26.0 (100.0)
Serum uric acid (mg/dL, X±SD)		7.09±1.59
Serum creatinine [mg/dL, median (IQR)]		1.03 (0.30)
CRP [mg/L, median (IQR)]		5.0 (11.0)
ESR [mm/h, median (IQR)]		12.0 (20.0)
		Rheumatoid arthritis group (n: 25)
Duration of diagnosis [months, median (IQR)]		66.0 (112.50)
Medication [n (%)]		
	sDMARDs	22.0 (88.0)
	bDMARDs	1.0 (4.0)
	sDMARDs+bDMARDs	2.0 (8.0)
Swollen joint count [median (IQR)]		0.0 (2.0)
Tender joint count [median (IQR)]		0.0 (2.0)
CRP [mg/L, median (IQR)]		4.50 (7.25)
ESR [mm/h, median (IQR)]		12.50 (18.50)
DAS-28 CRP [median (IQR)]		2.05 (1.30)
DAS-28 ESR [median (IQR)]		2.42 (1.55)

n: number; kg/m^2^: kilogram/meter square; IQR: interquartile range; X: mean; SD: standard deviation; sDMARDs: synthetic disease-modifying anti-rheumatic drugs; bDMARDs: biologic disease-modifying anti-rheumatic drugs; CRP: C-reactive protein; ESR: erythrocyte sedimentation rate; mg/dL: milligram/deciliter; mg/L: milligram/liter; mm/h: millimeter/hour; DAS-28: disease activity score.

The GS was similar between gout and HC (p>0.05). The dominant and non-dominant GE in gout was lower than in HC (p=0.008, p=0.001). The HC performed lower times for dominant (p=0.001, p<0.001, and p<0.001) and non-dominant (p=0.002, p=0.004, and p=0.002) placing, removing, and a total of NHPT than gout. Besides, RA had a higher non-dominant removing time than gout (p=0.009). The DHI score of gout was higher than HC (p=0.008) but lower than RA (p=0.002) (Table 2).

**Table 2:** Comparison of the hand function of groups.

Assessment parameters X±SD, median (IQR)		G1	G2	G3	P0	P1	P2	P3
Grip strength (kg-F)								
	DM	33.34±12.43	27.28±11.58	42.32±10.86	**<0.001** a *	0.202	0.023	**<0.001** **
	NDM	31.30±12.90	26.60±13.19	39.68±10.68	**0.001** a *	0.530	0.053	**0.001** **
Grip endurance (s)								
	DM	4.70 (5.92)	4.46 (4.97)	10.22 (4.65)	**<0.001** b *	0.277	**0.008** **	**<0.001** **
	NDM	5.44 (2.70)	4.48 (3.71)	8.92 (3.45)	**<0.001** b *	0.162	**0.001** **	**<0.001** **
Nine-Hole Peg Test (s)								
	DM placing time	18.75 (8.57)	17.94 (6.45)	15.09 (2.32)	**<0.001** b *	0.999	**0.001** **	**<0.001** **
	DM removing time	6.09 (1.52)	7.18 (3.57)	4.81 (2.01)	**<0.001** b *	0.051	**<0.001** **	**<0.001** **
	DM total time	24.86 (8.75)	25.30 (9.06)	19.44 (3.62)	**<0.001** b *	0.517	**<0.001** **	**<0.001** **
	NDM placing time	18.46 (5.49)	19.28 (2.78)	15.45 (3.10)	**<0.001** b *	0.311	**0.002** **	**<0.001** **
	NDM removing time	6.09 (1.74)	7.41 (1.81)	4.89 (1.65)	**<0.001** b *	**0.009** **	**0.004** **	**<0.001** **
	NDM total time	25.01 (7.25)	27.15 (3.71)	20.58 (4.14)	**<0.001** b *	0.058	**0.002** **	**<0.001** **
	DHI score	2.00 (6.00)	10.00 (15.00)	0 (0)	**<0.001** b *	**0.002** **	**0.008** **	**<0.001** **

X: mean; SD: standard deviation; IQR: interquartile range; n: number; DM: dominant; NDM: non-dominant; kg-F: kilogram-force; sec: second; DHI: Duruöz Hand Index; G1: gout group; G2: rheumatoid arthritis group; G3: non-arthritic control groups; P0: comparison of all groups; P1: comparison of gout and rheumatoid arthritis groups; P2: comparison of gout and non-arthritic control groups; P3: comparison of rheumatoid arthritis and non-arthritic control groups.

a One-Way ANOVA,

b Kruskal-Wallis test,

* p<0.050,

** p<0.016 (Bonferroni correction).

The significant values are represented in bold.

## DISCUSSION

We found that the GS of gout was similar to HC. However, GE was lower than in HC but like RA. Manual dexterity was worse in gout than in HC. It was as bad as RA, except for the non-dominant removal time. The perceived functional disability of the hand was higher in gout than in HC but lower than in RA. Patients with gout experience reduced GE, diminished manual dexterity, and increased functional impairment.

The GE was lower in participants with gout than HC. It was the first study to investigate GE in gout, but it was shown in other inflammatory arthritis. Köprülüoğlu et al. found that GE was lower in RA and psoriatic arthritis than HC^14^. Endurance is crucial for maintaining coordinated sensorimotor function. GE significantly impacts many daily activities, and its impairment can lead to difficulties in hand function^5,14,16^. Our research indicates that GE may also be impacted and lead to significant challenges in daily living in gout.

We investigated manual dexterity and found that gout had worse dexterity than HC. Additionally, except for the non-dominant removing time, it was as poor as RA. It was surprising that, although RA mainly affects the hand joints and worse manual dexterity was well-known^17^, participants with gout also experience manual dexterity impairments that are nearly equivalent to RA. It is an important issue that needs to be addressed. Manual dexterity involves skillful hand and arm movements that rely on strong sensorimotor functions that are important to perform daily living activities properly^18^. Therefore, the causes, including clinical, kinetic, and kinematic analyses of impairment in hand dexterity in gout, should be further investigated.

The perceived hand function was impaired in gout, although not as severely as in RA. Although previous studies mostly focus on foot function in daily living, they indicated that gout may cause problems with daily activities^19^. The impact of hand function on daily life can be understood from several perspectives. Structural changes in the hand joints and surrounding soft tissues that occur throughout the chronic progression of the disease can make it difficult for individuals to perform tasks that require hand function. Additionally, the burden of chronic disease, including repeated flares of gout and fatigue, can significantly affect a person's ability to participate in daily activities, leading to functional losses. As previously mentioned, GE and manual dexterity are key indicators of hand function^5,16,17^. The decreased GE and manual dexterity performance we observed may explain the expected finding. The results of our study highlight how these factors contribute to the functional limitations experienced by individuals with gout in their daily lives.

The limitation was that we didn't provide ultrasound or X-ray findings. This prevented us from assessing the extent of structural damage in the joints. Consequently, we could not adequately discuss our results on joint damage in patients with gout and RA. Future studies should explore the relationship between structural damage and hand function.

## CONCLUSION

It was the first study examining hand function in gout, comparing it with RA and HC. Patients with gout experience reduced GE, diminished manual dexterity, and increased functional impairment. Notably, the impairment in GE among gout patients is similar to that observed in RA patients. These results provide valuable insights into the characteristics of hand function in gout. Assessing hand function during clinical examinations of patients with gout should be considered. Therefore, it is clinically important to address these issues collaboratively with rheumatologists, physicians, and hand therapists.

## Data Availability

The datasets generated and/or analyzed during the current study are available from the corresponding author upon reasonable request.
